# Emerging trends in one- and two-dimensional nanomaterials

**DOI:** 10.1098/rsos.201786

**Published:** 2020-10-21

**Authors:** Nikos Tagmatarchis

**Affiliations:** Theoretical and Physical Chemistry Institute, National Hellenic Research Foundation, 48 Vassileos Constantinou Avenue, 11635 Athens, Greece

This themed collection of *Royal Society Open Science* manuscripts is dedicated to *Emerging trends in one- and two-dimensional nanomaterials* and features excellent contributions from world leaders in the field. Specifically, the issue deals with recent progress in the rapidly emerging topic of nanomaterials, focusing specifically on the synthesis, characterization and application of so-called nanocarbon materials (graphene, nanotubes and fullerenes). This timely collection of diverse research covers a wide variety of applications related to electronics, energy and quantum computing. Here, a brief overview of one- and two-dimensional nanomaterials is given, with some of the featured contributions highlighted with the aim of inspiring readers and stimulating further advances the field.

Carbon is the fourth most abundant element in the universe and carbon compounds form the basis of all known life on Earth. In the last few decades, new carbon allotropes, beyond diamond and graphite, have emerged, where carbon occupies diverse structures and configurations. The advent of one-dimensional carbon nanotubes (CNTs) by Iijima in 1991 [1], followed the discovery of zero-dimensional fullerenes by Kroto *et al*. [2], and the field was further enriched with the isolation of two-dimensional graphene by Novoselov & co-workers [3]. It is without doubt that nanocarbon structures have received significant attention, particularly because they can be used to test fundamental ideas about the role of dimensionality and confinement in materials.

The vast majority of contributions to this issue are devoted to carbon-based nanomaterials. In this context, the energetics of perylene encapsulated within a metallic (11,11) CNT and a semiconducting (19,0) CNT were computationally examined by Nagasawa *et al*. [4]. The authors find that a flat molecular conformation of the encapsulated perylene moieties energetically competes with stacking, while the electronic structure of the CNTs is weakly affected by the orientation of the perylene molecules. In another theoretical paper, Edvinsson [5] has studied the effect on the optical and photocatalytic properties of the direct band and indirect band gap semiconductor materials, based on known equations for optical transitions and carrier confinement. An experimental examination of the floating catalyst chemical vapour deposition synthesis of single-walled CNTs for high-performance transparent conducting films is presented by Ding *et al*. [6].

Recent progress in developing copper/CNT composites towards the realization of lighter alternatives to copper is reviewed by Sekiguchi & co-workers [7]. The authors discuss and indicate methods to develop Cu/CNT composites with better electrical performance as compared to Cu. Next-generation applications for the electronics industry are proposed. On a different note, but still on the subject of one-dimensional CNTs, Gao & Kono [8] review recent discoveries in optical spectroscopy and optoelectronic device applications with films of CNTs obtained through a controlled vacuum filtration technique.

Transmission electron microscopy (TEM) imaging has been employed to monitor nanosized particles and CNT growth in a statistical manner by Xiang and Maruyama [9]. Further TEM imaging was employed by Pérez-Luna *et al.* [10] to locally probe the presence of functional groups covalently attached to CNTs and graphene. On the topic of inorganic two-dimensional nanomaterials, Virkki *et al.* [11] present results from the fabrication of ZnO nanorods and have examined their sensitization by an aggregation-protected phthalocyanine derivative through transient absorption spectroscopy.

Finally, on the subject of fullerenes, Macpherson *et al.* [12] have achieved the synthesis of a N@C_60_-containing C_60_ tetramer based on the quadruple 1,3-dipolar cycloaddition reaction, working towards the realization of a scaffold for measuring multiple qubit–qubit interactions. A further paper on fullerenes by Aoyagi *et al.* [13] reports on the structure of crystalline C_60_ with Li^+^ inside the cage. The work aids our understanding of the electrostatic and thermal properties of an encapsulated lithium cation. The isolation of CF_3_-functionalized Gd@C_74_ and its structural characterization based on single-crystal X-ray diffraction is reported by Nakagawa & co-workers [14]

The guest co-editors of this themed issue, Professor Hisanori Shinohara, at the Department of Chemistry, Nagoya University, Japan; Professor Young Hee Lee, at the Department of Energy Science & Department of Physics, Sungkyunkwan University, Korea; and Dr Nikos Tagmatarchis, at the Theoretical and Physical Chemistry Institute, National Hellenic Research Foundation, Greece, wish to thank all contributors for their enthusiasm to participate. We would also like to express our gratitude to the Royal Society of Chemistry team and in particular for the staff of *Royal Society Open Science* for their continuous support for the realization of this issue devoted to *Emerging trends in one- and two-dimensional nanomaterials*.

## Supplementary Material

Reviewer comments

## Data accessibility

This article has no additional data.
