# Supplementary material for: Emerging trends in one- and two-dimensional nanomaterials
Source: R Soc Open Sci. 2020 Oct 21;7(10):201786. doi: 10.1098/rsos.201786 (PMC7657888; doi:10.1098/rsos.201786)
Supplement: Reviewer comments [file rsos201786_review_history.pdf]

THE ROYAL SOCIETY
PUBLISHING

ROYAL SOCIETY
OPEN SCIENCE

# Emerging trends in one- and two-dimensional nanomaterials

Nikos Tagmatarchis

**Article citation details**
*R. Soc. open sci.* **7**: 201786.
http://dx.doi.org/10.1098/rsos.201786

**Review timeline**
Original submission: 6 October 2020
Final acceptance: 6 October 2020

Note: Reports are unedited and appear as submitted by the referee. The review history appears in chronological order.

# Review History

## Decision letter (RSOS-201786.R0)

We hope you are keeping well at this difficult and unusual time. We continue to value your support of the journal in these challenging circumstances. If Royal Society Open Science can assist you at all, please don't hesitate to let us know at the email address below.

Dear Dr Tagmatarchis,

I am pleased to inform you that your manuscript entitled "Emerging trends in one- and two-dimensional nanomaterials" is now accepted for publication in Royal Society Open Science.

You can expect to receive a proof of your article in the near future. Please contact the editorial office (openscience_proofs@royalsociety.org) and the production office (openscience@royalsociety.org) to let us know if you are likely to be away from e-mail contact -- if you are going to be away, please nominate a co-author (if available) to manage the proofing process, and ensure they are copied into your email to the journal.

Due to rapid publication and an extremely tight schedule, if comments are not received, your paper may experience a delay in publication.

Royal Society Open Science operates under a continuous publication model. Your article will be published as soon as it is ready for publication, and this will be the final version of the paper. As such, it can be cited immediately by other researchers. As the issue version of your paper will be

Reports © 2020 The Reviewers; Decision Letters © 2020 The Reviewers and Editors; Responses © 2020 The Reviewers, Editors and Authors. Published by the Royal Society under the terms of the Creative Commons Attribution License http://creativecommons.org/licenses/by/4.0/, which permits unrestricted use, provided the original author and source are credited

the only version to be published I would advise you to check your proofs thoroughly as changes cannot be made once the paper is published.

Please see the Royal Society Publishing guidance on how you may share your accepted author manuscript at https://royalsociety.org/journals/ethics-policies/media-embargo/.

On behalf of the Editors of Royal Society Open Science, we look forward to your continued contributions to the Journal.

Kind regards,
Andrew Dunn
Royal Society Open Science Editorial Office
Royal Society Open Science
openscience@royalsociety.org

Follow Royal Society Publishing on Twitter: @RSocPublishing
Follow Royal Society Publishing on Facebook:
https://www.facebook.com/RoyalSocietyPublishing.FanPage/
Read Royal Society Publishing's blog:
https://royalsociety.org/blog/blogsearchpage/?category=Publishing
